# Bailey pairs and quantum *q*-series identities. I. The classical identities

**DOI:** 10.1007/s40065-025-00594-0

**Published:** 2025-11-26

**Authors:** Jehanne Dousse, Jeremy Lovejoy

**Affiliations:** 1 Université de Genève, 7-9, rue Conseil Général, 1205 Geneva, Switzerland; 2 CNRS, Université Paris Cité, Bâtiment Sophie Germain, Case Courier 7014, 8 Place Aurélie Nemours, 75205 Paris Cedex 13, France

**Keywords:** 33D15

## Abstract

We use Bailey pairs to prove *q*-series identities at roots of unity due to Cohen and Bryson–Ono–Pitman–Rhoades. The proofs use Bailey pairs with quadratic forms developed in the study of mock theta functions. In addition to the standard Bailey lemma, we require some changes-of-base established by Bressoud–Ismail–Stanton. We then embed the identities in infinite families using the Bailey chain.

## Introduction

Recall the standard *q*-series notation,

$$\begin{aligned} (a)_n = (a;q)_n = \prod _{k=0}^{n-1} (1-aq^k), \end{aligned}$$

valid for non-negative integers *n*. In a study of *q*-series from Ramanujan’s lost notebook, Cohen [7] proved that for any root of unity *q*, 

1.1 $$\begin{aligned} \sum _{n \ge 0} (-1)^n(q^{-1};q^{-1})_n = \sum _{n \ge 0} (q^2;q^2)_{n}q^{n+1}. \end{aligned}$$

This is not an identity between complex functions in any classical sense. But at any root of unity $q = \xi$ both series truncate, leaving a polynomial identity. For example at $q=i$ the left-hand side is

$$\begin{aligned} 1 - (1+i)+ (1+i)(1-i^2) - (1+i)(1-i^2)(1+i^3) = -2+i, \end{aligned}$$

while the right-hand side is

$$\begin{aligned} i + i^2(1-i^2) = -2+i. \end{aligned}$$

Cohen also left two similar identities as exercises for the reader, 
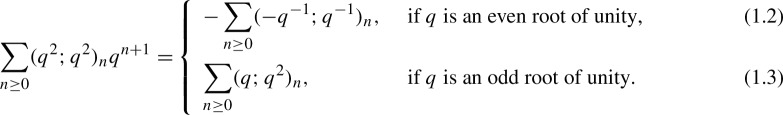
 Later, in a study of quantum modular forms, Bryson et al. [6] proved another elegant identity at roots of unity,

1.4 $$\begin{aligned} \sum _{n \ge 0} (q^{-1};q^{-1})_n = \sum _{n \ge 0} (q)_n^2q^{n+1}. \end{aligned}$$

These early examples of Cohen and Bryson–Ono–Pitman–Rhoades laid the foundation for future work on *q*-series identities at roots of unity, also known as *quantum q-series identities*. While the original proofs are based on elementary recursions, the identities can also be deduced using classical *q*-series transformations, as can a great many other such identities [11]. The identity (1.4) can also be deduced using the colored Jones polynomial of the trefoil knot [8], and this observation led to many further families of identities at roots of unity, including several generalizations of (1.4) [8, 12, 13]. Quantum *q*-series identities also arise as a consequence of so-called “strange identities” for quantum modular forms [10].

In this paper we show how Bailey pairs may be used to prove (1.1)–(1.4). Our proofs use Bailey pairs with quadratic forms developed in the study of mock theta functions. We use these pairs to express the relevant *q*-series as truncated indefinite theta series. See Propositions 3.1–3.4. Surprisingly, for two of the four identities a key role is played by changes-of-base for Bailey pairs due to Bressoud–Ismail–Stanton [5].

Once we have understood the identities using Bailey pairs, they can be embedded in infinite families using the machinery of the Bailey chain. For example, we will show the following.

### Theorem 1.1

For $m \ge 1$ and *q* any root of unity we have

1.5 $$\begin{aligned}  &   \sum _{n_m \ge \cdots \ge n_1 \ge 0} (q)_{n_m}(-1)^{n_1}q^{-\left( {\begin{array}{c}n_1+1\\ 2\end{array}}\right) }\prod _{i=1}^{m-1} q^{n_i^2+n_i} \begin{bmatrix} n_{i+1} \\ n_i \end{bmatrix} \nonumber \\  &   \quad = \sum _{n_m \ge \cdots \ge n_1 \ge 0} (q)_{n_m}(q)_{n_1}q^{n_m+1}\prod _{i=1}^{m-1} q^{n_i^2+n_i} \begin{bmatrix} n_{i+1} \\ n_i \end{bmatrix}. \end{aligned}$$

### Theorem 1.2

For $m \ge 1$ and *q* any even root of unity we have

1.6 $$\begin{aligned}  &   \sum _{n_m \ge \cdots \ge n_1 \ge 0} (-q)_{n_m}(q)_{n_1}q^{n_m+1}\prod _{i=1}^{m-1} q^{n_i^2+n_i} \begin{bmatrix} n_{i+1} \\ n_i \end{bmatrix} \nonumber \\  &   \quad = -\sum _{n_m \ge \cdots \ge n_1 \ge 0} (-q)_{n_m}(-1)^{n_m+ n_1}q^{- \left( {\begin{array}{c}n_1+1\\ 2\end{array}}\right) }\prod _{i=1}^{m-1} q^{n_i^2+n_i} \begin{bmatrix} n_{i+1} \\ n_i \end{bmatrix}. \end{aligned}$$

Here we have used the *q*-binomial coefficient (or Gaussian polynomial),

$$\begin{aligned} \begin{bmatrix} n \\ k \end{bmatrix} = \begin{bmatrix} n \\ k \end{bmatrix}_q = {\left\{ \begin{array}{ll} \frac{(q)_n}{(q)_{n-k}(q)_k}, &  \text {if } 0 \le k \le n, \\ 0, &  \text {otherwise}. \end{array}\right. } \end{aligned}$$

Note that the cases $m=1$ of (1.5) and (1.6) are (1.4) and (1.2), respectively. This follows from the relation

1.7 $$\begin{aligned} (q^{-1};q^{-1})_n = (q)_n(-1)^nq^{-\left( {\begin{array}{c}n+1\\ 2\end{array}}\right) }. \end{aligned}$$

Generalizations of (1.1) and (1.3) are contained in the following two results. Here the *q*-series do not always truncate naturally, so the multisums come with an upper bound.

### Theorem 1.3

For $m \ge 1$ and *q* any primitive *N*th root of unity we have

$$\begin{aligned} \begin{aligned}&\sum _{N-1 \ge n_{2m-1} \ge \cdots \ge n_1 \ge 0} (-q^{n_1+1})_{n_{2m-1}-n_1} (-1)^{n_{2m-1}} (q)_{n_1} \prod _{i=1}^{2m-2} q^{n_i^2+n_i} \begin{bmatrix} n_{i+1} \\ n_i \end{bmatrix} \\&\quad = \sum _{N-1 \ge n_m \ge \cdots \ge n_1 \ge 0} (-q^{2n_1+2})_{2n_m-2n_1}(-1)^{n_m}q^{-(n_m+1)^2}(q^2;q^2)_{n_1} \prod _{i=1}^{m-1} q^{2n_i^2+2n_i} \begin{bmatrix} n_{i+1} \\ n_i \end{bmatrix}_{q^2}. \end{aligned} \end{aligned}$$

### Theorem 1.4

For $m \ge 1$ and *q* any primitive odd *N*th root of unity we have

$$\begin{aligned} \begin{aligned}&\sum _{N-1 \ge n_m \ge \cdots \ge n_1 \ge 0} (-q^{2n_1+2})_{2n_m-2n_1}(-1)^{n_m}q^{-(n_m+1)^2}(q^2;q^2)_{n_1} \prod _{i=1}^{m-1} q^{2n_i^2+2n_i} \begin{bmatrix} n_{i+1} \\ n_i \end{bmatrix}_{q^2} \\&\quad = \sum _{N-1 \ge n_m \ge \cdots \ge n_1 \ge 0} (-q^{2n_1+1})_{2n_m-2n_1}(-1)^{n_m}q^{-n_m^2}(q;q^2)_{n_1} \prod _{i=1}^{m-1} q^{2n_i^2+2n_i} \begin{bmatrix} n_{i+1} \\ n_i \end{bmatrix}_{q^2}. \end{aligned} \end{aligned}$$

Note that when $m=1$ and $q=1/q$ Theorems 1.3 and 1.4 reduce to (1.1) and (1.3), respectively.

The rest of the paper is organized as follows. In the next section we review the classical Bailey lemma along with some changes-of-base. In Sect. 3 we prove the classical quantum *q*-series identities in (1.1)–(1.4). In Sect. 4 we prove Theorems 1.1–1.4. Future papers in this series will be devoted to a more thorough investigation of the role Bailey pairs play in proving quantum *q*-series identities as well as the role such identities play in establishing the quantum modularity of the relevant series. We say a little more about this in Sect. 5.

## The Bailey lemma

We begin by reviewing some basic facts about Bailey pairs. For more background, see [1, 3, 14]. A pair of sequences $(\alpha _n,\beta _n)$ is a Bailey pair relative to *a* if

2.1 $$\begin{aligned} \beta _n&= \sum _{k=0}^n \frac{\alpha _k}{(q)_{n-k}(aq)_{n+k}} \end{aligned}$$

2.2 $$\begin{aligned}&= \frac{1}{(q)_n(aq)_n} \sum _{k=0}^n \frac{(q^{-n})_k}{(aq^{n+1})_k}(-1)^kq^{nk -\left( {\begin{array}{c}k\\ 2\end{array}}\right) } \alpha _k. \end{aligned}$$

Equation (2.1) is the original definition, while (2.2) follows using the relation

2.3 $$\begin{aligned} (x)_{n-k} = \frac{(x)_n}{(q^{1-n}/x)_k}(-q/x)^kq^{\left( {\begin{array}{c}k\\ 2\end{array}}\right) - nk}. \end{aligned}$$

The following, known as the Bailey lemma and due to Andrews [1], produces new Bailey pairs from a given pair.

### Lemma 2.1

If $(\alpha _n,\beta _n)$ is a Bailey pair relative to *a*,  then so is $(\alpha _n',\beta _n'),$ where

2.4 $$\begin{aligned} \alpha _n' = \frac{(b)_n(c)_n (aq/bc)^n}{(aq/b)_n(aq/c)_n} \alpha _n \end{aligned}$$

and

2.5 $$\begin{aligned} \beta _n'&= \frac{1}{(aq/b,aq/c)_n} \sum _{k=0}^n \frac{(b)_k(c)_k(aq/bc)_{n-k} (aq/bc)^k}{(q)_{n-k}} \beta _k \end{aligned}$$

2.6 $$\begin{aligned}&= \frac{(aq/bc)_n}{(q)_n(aq/b)_n(aq/c)_n} \sum _{k=0}^n \frac{(b)_k(c)_k(q^{-n})_kq^k}{(bcq^{-n}/a)_k}\beta _k . \end{aligned}$$

Repeated application of the Bailey lemma is called the Bailey chain. We record the cases $b,c \rightarrow \infty$ and $b,c \rightarrow 0$ of (2.5) with $a=q.$

### Lemma 2.2

If $(\alpha _n,\beta _n)$ is a Bailey pair relative to *q*,  then so is $(\alpha _n', \beta _n'),$ where

$$\begin{aligned} \alpha _n' = q^{n^2+n}\alpha _n \end{aligned}$$

and

$$\begin{aligned} \beta _n' = \sum _{k=0}^n \frac{q^{k^2+k}}{(q)_{n-k}}\beta _k. \end{aligned}$$

### Lemma 2.3

If $(\alpha _n,\beta _n)$ is a Bailey pair relative to *q*,  then so is $(\alpha _n', \beta _n'),$ where

$$\begin{aligned} \alpha _n' = q^{-n^2-n}\alpha _n \end{aligned}$$

and

$$\begin{aligned} \beta _n' = (-1)^nq^{-\left( {\begin{array}{c}n+1\\ 2\end{array}}\right) -n}\sum _{k=0}^n \frac{q^{\left( {\begin{array}{c}k+1\\ 2\end{array}}\right) -nk}(-1)^k}{(q)_{n-k}}\beta _k. \end{aligned}$$

Using (2.4) and (2.6) in (2.2) with $n=n-1$ and $a=q$ gives a key identity, which we state as a lemma.

### Lemma 2.4

If $(\alpha _n,\beta _n)$ is a Bailey pair relative to *q*,  then we have

$$\begin{aligned} \frac{(q^2/bc)_{n-1}(q^2)_{n-1}}{(q^2/b)_{n-1}(q^2/c)_{n-1}}\sum _{k=0}^{n-1} \frac{(b)_k(c)_k(q^{1-n})_kq^k}{(bcq^{-n})_k}\beta _k = \sum _{k=0}^{n-1} \frac{(q^{1-n})_k(b)_k(c)_kq^{nk-\left( {\begin{array}{c}k+1\\ 2\end{array}}\right) } (-q^2/bc)^k}{(q^{1+n})_k(q^2/b)_k(q^2/c)_k}\alpha _k. \end{aligned}$$

Next, we record two changes-of-base for Bailey pairs due to Bressoud, Ismail, and Stanton [5]. The first is the case $a=q$ of [5, D(1)] while the second is the case $a=q$ of [5, D(4)].

### Lemma 2.5

If $(\alpha _n,\beta _n)$ is a Bailey pair relative to *q*,  then so is $(\alpha _n', \beta _n'),$ where

$$\begin{aligned} \alpha _n' = \alpha _n(q^2), \end{aligned}$$

and

$$\begin{aligned} \beta _n'= \sum _{k=0}^n \frac{(-q^2)_{2k}}{(q^2;q^2)_{n-k}}q^{n-k}\beta _k(q^2). \end{aligned}$$

### Lemma 2.6

If $(\alpha _n,\beta _n)$ is a Bailey pair relative to *q*,  then so is $(\alpha _n', \beta _n'),$ where

$$\begin{aligned} \alpha _n' = \frac{1+q}{1+q^{2n+1}}q^n\alpha _n(q^2), \end{aligned}$$

and

$$\begin{aligned} \beta _n' = \sum _{k=0}^n \frac{(-q)_{2k}}{(q^2;q^2)_{n-k}}q^k\beta _k(q^2). \end{aligned}$$

Using these in (2.2) and rewriting using (2.3) gives two more key identities.

### Lemma 2.7

If $(\alpha _n,\beta _n)$ is a Bailey pair relative to *q*,  then

$$\begin{aligned} \begin{aligned}&\sum _{k=0}^{n-1}(-q^2)_{2k}(q^{2-2n};q^2)_k(-1)^kq^{n(2k+1)-k^2-2k-1} \beta _k(q^2) \\&\quad = \frac{(-q)_{n-1}}{(q^2)_{n-1}}\sum _{k=0}^{n-1} \frac{(q^{1-n})_k}{(q^{1+n})_k}(-1)^kq^{nk-\left( {\begin{array}{c}k+1\\ 2\end{array}}\right) } \alpha _k(q^2). \end{aligned} \end{aligned}$$

### Lemma 2.8

If $(\alpha _n,\beta _n)$ is a Bailey pair relative to *q*,  then

$$\begin{aligned} \begin{aligned}&\sum _{k=0}^{n-1}(-q)_{2k}(q^{2-2n};q^2)_k(-1)^kq^{2nk-k^2} \beta _k(q^2) \\&\quad = \frac{(-q)_{n-1}}{(q^2)_{n-1}}\sum _{k=0}^{n-1} \frac{(q^{1-n})_k(1+q)}{(q^{1+n})_k(1+q^{2k+1})}(-1)^kq^{nk-\left( {\begin{array}{c}k\\ 2\end{array}}\right) } \alpha _k(q^2). \end{aligned} \end{aligned}$$

Finally, we note five Bailey pairs.

### Lemma 2.9

The following are Bailey pairs relative to *q*.

2.7 $$\begin{aligned} \alpha _k&= \frac{(1-q^{2k+1})q^{-k}}{1-q}\sum _{j=-k}^k(-1)^jq^{j(3j+1)/2} \ \ \ \text {and} \ \ \ \beta _k = \frac{q^{-k}}{(q)_k}, \end{aligned}$$

2.8 $$\begin{aligned} \alpha _k&= \frac{(1-q^{2k+1})}{1-q}q^{2k^2+k}\sum _{j=-k}^k (-1)^jq^{-j(3j+1)/2} \ \ \ \text {and } \ \ \ \beta _k = 1, \end{aligned}$$

2.9 $$\begin{aligned} \alpha _k&= \frac{(1-q^{2k+1})}{1-q}q^{k(3k+1)/2}\sum _{j = -k}^k (-1)^jq^{-j^2} \ \ \ \text {and} \ \ \ \beta _k = \frac{1}{(-q)_k}, \end{aligned}$$

2.10 $$\begin{aligned} \alpha _k&= \frac{(1-q^{k+1/2})}{1-q^{1/2}}q^{k^2+k/2}\sum _{j=-k}^k (-1)^jq^{-j^2/2} \ \ \ \text {and} \ \ \ \beta _k = \frac{1}{(-q;q^{1/2})_{2k}}, \end{aligned}$$

2.11 $$\begin{aligned} \alpha _k&= \frac{(1-q^{2k+1})}{(1-q)} q^{k^2} \sum _{j=-k}^k (-1)^j q^{-j^2/2} \ \ \ \text {and} \ \ \ \beta _k = \frac{(q^{1/2};q)_k}{(q)_k(-q^{1/2};q^{1/2})_{2k}}. \end{aligned}$$

### Proof

The Bailey pairs (2.8) and (2.9) are due to Andrews [2], while the pair (2.10) arose in work of Andrews and Hickerson [4]. The remaining pairs are easily deduced from [9, Theorem 7]. Namely, (2.7) is the case $b,c,d \rightarrow \infty$ and (2.11) is the case $b=-1,$ $c=-q^{1/2},$ and $d=0.$ $\square$

## Proofs of (1.1)–(1.4)

In this section we prove the quantum *q*-series identities in (1.1)–(1.4). In each case we use Bailey pairs to express both sides of the identity as truncated indefinite theta series. We begin with (1.4), which will follow immediately from the next proposition. We use the standard convention that for any *f*, 

$$\begin{aligned} \sum _{j=a}^b f(j) = - \sum _{j=b+1}^{a-1} f(j) \end{aligned}$$

whenever $$a > b.$$

### Proposition 3.1

For *q* a primitive *n*th root of unity we have

3.1 $$\begin{aligned} \sum _{k=0}^{n-1} (q)_k&= -\frac{1}{n} \sum _{k=-n}^{n-1} \sum _{j=-k}^k (k^2-j(3j+1)/2) q^{-k^2 + j(3j+1)/2}(-1)^j, \end{aligned}$$

3.2 $$\begin{aligned} \sum _{k=0}^{n-1} (q)_k^2q^{k+1}&= -\frac{1}{n} \sum _{k=-n}^{n-1} \sum _{j=-k}^k (k^2-j(3j+1)/2)q^{k^2-j(3j+1)/2)}(-1)^j. \end{aligned}$$

### Proof

We begin with (3.1). Letting $b \rightarrow 0$ and setting $c=q$ in Lemma 2.4 we have that if $(\alpha _k,\beta _k)$ is a Bailey pair relative to *q*,  then

3.3 $$\begin{aligned} \frac{1-q^n}{1-q}\sum _{k =0}^{n-1} (q)_k(q^{1-n})_kq^{k+1} \beta _k = q^n\sum _{k=0}^{n-1} \frac{(q^{1-n})_k}{(q^{1+n})_k}q^{nk - (k^2+k)}\alpha _k. \end{aligned}$$

Using the Bailey pair (2.7) in (3.3) we have the rational function identity

$$\begin{aligned} (1-q^n)\sum _{k =0}^{n-1} (q^{1-n})_k = q^n \sum _{k=0}^{n-1} \frac{(q^{1-n})_k}{(q^{1+n})_k}q^{nk-k^2-2k-1}(1-q^{2k+1})\sum _{j=-k}^k(-1)^jq^{j(3j+1)/2}. \end{aligned}$$

In light of the factor $(1-q^n)$ on the left-hand side, we see that the right-hand side vanishes when *q* is an *n*th root of unity. Using l’Hôpital’s rule we obtain that if $q = \zeta _n$ is a primitive *n*th root of unity, then

$$\begin{aligned} \sum _{k=0}^{n-1} (q)_k&= \lim _{q \rightarrow \zeta _n} \frac{1}{1-q^n} \sum _{k=0}^{n-1} q^{-k^2-2k-1}(1-q^{2k+1})\sum _{j=-k}^k (-1)^jq^{j(3j+1)/2}\\  &= -\frac{q}{n} \frac{d}{dq}\Big |_{q= \zeta _n} \sum _{k=0}^{n-1} q^{-k^2-2k-1}(1-q^{2k+1})\sum _{j=-k}^k (-1)^jq^{j(3j+1)/2} \\  &= \frac{q}{n} \frac{d}{dq}\Big |_{q= \zeta _n} \sum _{k=-n}^{n-1}\sum _{j=-k}^k q^{-k^2+j(3j+1)/2}(-1)^j, \end{aligned}$$

and (3.1) follows. In the above we have used the fact that for *q* a primitive *n*th root of unity and $0 \le k \le n-1,$

$$\begin{aligned} \frac{(q^{1-n})_k}{(q^{1+n})_k} =1. \end{aligned}$$

For (3.2), we use (2.8) in (3.3) and perform a similar calculation. This completes the proof. $\square$

Next we treat (1.2). This follows immediately from the next proposition. We use the sign function

$$\begin{aligned} \textrm{sgn}(k) = {\left\{ \begin{array}{ll} 1, &  \text {if } k \ge 0, \\ -1, &  \text {if } k < 0. \end{array}\right. } \end{aligned}$$

### Proposition 3.2

If *q* is a primitive *n*th root of unity we have

3.4 $$\begin{aligned} \sum _{k=0}^{n-1} (q^2;q^2)_kq^{k+1} = {\left\{ \begin{array}{ll} -\frac{1}{n^2} \mathop {\mathop {\sum }\limits _{{k=-n}}}\limits ^{{n-1}} \mathop {\mathop {\sum }\limits _{{j=-k}}}\limits ^{k} \text {sgn}(k) (k^2-j(3j+1)/2)(-1)^{k+j}q^{k^2-j(3j+1)/2}, \ n \text{ odd } , \\ -\frac{1}{4} \mathop {\mathop {\sum }\limits _{{k=-n}}}\limits ^{{n-1}} \mathop {\mathop {\sum }\limits _{{j=-k}}}\limits ^{k} \text {sgn}(k)(-1)^{k+j}q^{k^2-j(3j+1)/2}, \ n \text{ even } , \end{array}\right. } \end{aligned}$$

and if *q* is a primitive even *n*th root of unity then

3.5 $$\begin{aligned} \sum _{k=0}^{n-1} (-q)_k = \frac{1}{4} \sum _{k=-n}^{n-1} \sum _{j=-k}^k \textrm{sgn}(k)(-1)^{k+j}q^{-k^2+j(3j+1)/2}. \end{aligned}$$

### Proof

If we take $b \rightarrow 0$ and set $c=-q$ in Lemma 2.4 we have that if $(\alpha _k,\beta _k)$ is a Bailey pair relative to *q*,  then

3.6 $$\begin{aligned} \sum _{k =0}^{n-1} (-q)_k(q^{1-n})_kq^{k+1} \beta _k = -(-q)^{n}\frac{(-q)_{n-1}}{(q^2)_{n-1}}\sum _{k=0}^{n-1} \frac{(q^{1-n})_k}{(q^{1+n})_k}q^{nk - (k^2+k)}(-1)^k\alpha _k. \end{aligned}$$

Using the Bailey pair (2.8) gives the rational function identity

$$\begin{aligned} \begin{aligned}&\sum _{k =0}^{n-1} (-q)_k(q^{1-n})_kq^{k+1} \\&\quad = -(-q)^{n}\frac{(-q)_{n-1}}{(q)_{n}}\sum _{k=0}^{n-1} \frac{(q^{1-n})_k}{(q^{1+n})_k}q^{nk +k^2}(-1)^k (1-q^{2k+1})\sum _{j=-k}^k (-1)^jq^{-j(3j+1)/2}. \end{aligned} \end{aligned}$$

We first consider the case when *q* is a primitive odd *n*th root of unity $\zeta _n.$ In this case, we have $(-q)_{n-1} = 1$ and $(q)_{n-1}=n.$ Hence we have

$$\begin{aligned} \sum _{k=0}^{n-1} (q^2;q^2)_{k}q^{k+1}&= \lim _{q \rightarrow \zeta _n} \frac{1}{n(1-q^n)} \sum _{k=0}^{n-1}\sum _{j=-k}^k (-1)^{j+k}q^{k^2-j(3j+1)/2}(1-q^{2k+1}) \\  &= -\frac{q}{n^2} \frac{d}{dq}\Big |_{q = \zeta _n} \sum _{k=-n}^{n-1}\sum _{j=-k}^k \text {sgn}(k)(-1)^{j+k}q^{k^2-j(3j+1)/2}. \end{aligned}$$

Equation (3.4) follows for *n* odd.

Next assume that *n* is even. In this case, we still have $(q)_{n-1} = n$ but now $(-q)_{n-1}=0.$ A short calculation gives that for a primitive even *n*th root of unity $\zeta _n,$ we have $\frac{d}{dq}\big |_{q=\zeta _n} (-q)_{n-1} = n^2/4\zeta _n.$ With this in mind, for $q = \zeta _n$ we obtain

$$\begin{aligned} \sum _{k=0}^{n-1} (q^2;q^2)_{k}q^{k+1}&= \lim _{q \rightarrow \zeta _n} \frac{-(-q)_{n-1}}{n(1-q^n)} \sum _{k=0}^{n-1}\sum _{j=-k}^k (-1)^{j+k}q^{k^2-j(3j+1)/2}(1-q^{2k+1}) \\&= \frac{1}{4} \sum _{k=-n}^{n-1}\sum _{j=-k}^k \textrm{sgn}(k)(-1)^{j+k}q^{k^2-j(3j+1)/2}. \end{aligned}$$

This completes the proof of (3.4). Equation (3.5) follows in a similar manner using the Bailey pair (2.7) in (3.6). In obtaining the left-hand side of (3.5) from (3.6) we use the fact that if *q* is a primitive *n*th root of unity, then for $0 \le k \le n-1$ we have

$$\begin{aligned} \frac{(q^{1-n})_k}{(q)_k} =1. \end{aligned}$$

$$\square $$

We now move on to (1.1), which is an immediate consequence of the following proposition.

### Proposition 3.3

For *q* a primitive *n*th root of unity we have

3.7 $$\begin{aligned} \sum _{k=0}^{n-1} (q)_k(-1)^k = {\left\{ \begin{array}{ll} -\frac{1}{n^2} \mathop {\mathop {\sum }\limits _{{k=-n}}}\limits ^{{n-1}} \mathop {\mathop {\sum }\limits _{{j=-k}}}\limits ^{k} \text {sgn}(k) (k(3k+1)/2 - j^2)(-1)^{k+j}q^{k(3k+1)/2 - j^2}, \ n \text{ odd } , \\ \frac{1}{4} \mathop {\mathop {\sum }\limits _{{k=-n}}}\limits ^{{n-1}} \mathop {\mathop {\sum }\limits _{{j=-k}}}\limits ^{k} \text {sgn}(k)(-1)^{k+j}q^{k(3k+1)/2 - j^2}, \ n \text{ even } , \end{array}\right. } \end{aligned}$$

and

3.8 $$\begin{aligned} \begin{aligned}&\sum _{k=0}^{n-1} (q^2;q^2)_kq^{k+1} \\  &\quad = {\left\{ \begin{array}{ll} -\frac{1}{n^2} \mathop {\mathop {\sum }\limits _{{k=-n}}}\limits ^{{n-1}} \mathop {\mathop {\sum }\limits _{{j=-k}}}\limits ^{k} \text {sgn}(k) (k(3k+1)/2 - j^2)(-1)^{k+j}q^{-k(3k+1)/2 + j^2}, \ n \text{ odd } , \\ \frac{1}{4} \mathop {\mathop {\sum }\limits _{{k=-n}}}\limits ^{{n-1}} \mathop {\mathop {\sum }\limits _{{j=-k}}}\limits ^{k} \text {sgn}(k)(-1)^{k+j}q^{-k(3k+1)/2 + j^2}, \ n \text{ even } . \end{array}\right. } \end{aligned} \end{aligned}$$

### Proof

Applying Lemma 2.4 with $b \rightarrow \infty$ and $c=-q,$ we find that if $(\alpha _n,\beta _n)$ is a Bailey pair relative to *q*,  then

3.9 $$\begin{aligned} \sum _{k =0}^{n-1} (-q)_k(q^{1-n})_k(-1)^kq^{nk} \beta _k = \frac{(-q)_{n-1}}{(q^2)_{n-1}}\sum _{k=0}^{n-1} \frac{(q^{1-n})_k}{(q^{1+n})_k}q^{nk}(-1)^k\alpha _k. \end{aligned}$$

If we use the Bailey pair (2.9) and argue as usual, we obtain (3.7).

Now we take Lemma 2.7 and insert the Bailey pair (2.10) to obtain the identity

$$\begin{aligned} \begin{aligned}&\sum _{k=0}^{n-1}(q^{2-2n};q^2)_k(-1)^kq^{n(2k+1)-k^2-2k-1} \\&\quad = \frac{(-q)_{n-1}}{(q)_{n}}\sum _{k=0}^{n-1} \frac{(q^{1-n})_k}{(q^{1+n})_k}(-1)^kq^{nk+k(3k+1)/2} (1-q^{2k+1}) \sum _{j=-k}^k (-1)^jq^{-j^2}. \end{aligned} \end{aligned}$$

If *q* is a primitive *n*th root of unity, this leads to

$$\begin{aligned} \begin{aligned}&\sum _{k=0}^{n-1} (q^2;q^2)_k(-1)^kq^{-k^2-2k-1} \\  &\quad = {\left\{ \begin{array}{ll} -\frac{1}{n^2} \mathop {\mathop {\sum }\limits _{{k=-n}}}\limits ^{{n-1}} \mathop {\mathop {\sum }\limits _{{j=-k}}}\limits ^{k} \text {sgn}(k) (k(3k+1)/2 - j^2)(-1)^{k+j}q^{k(3k+1)/2 - j^2}, \ n \text{ odd } , \\ \frac{1}{4} \mathop {\mathop {\sum }\limits _{{k=-n}}}\limits ^{{n-1}} \mathop {\mathop {\sum }\limits _{{j=-k}}}\limits ^{k} \text {sgn}(k)(-1)^{k+j}q^{k(3k+1)/2 - j^2}, \ n \text{ even } . \end{array}\right. } \end{aligned} \end{aligned}$$

Replacing *q* by $q^{-1}$ and using (1.7) gives (3.8). $\square$

We conclude with (1.3), which will follow from the proposition below combined with (3.8).

### Proposition 3.4

If *q* is a primitive odd *n*th root of unity,  we have

$$\begin{aligned} \sum _{n \ge 0} (q;q^2)_n = -\frac{1}{n^2} \sum _{k=-n}^{n-1} \sum _{j=-k}^k \text {sgn}(k) (k(3k+1)/2 - j^2)(-1)^{k+j}q^{-k(3k+1)/2 + j^2}. \end{aligned}$$

### Proof

Inserting (2.11) into Lemma 2.8 gives that for *q* any primitive *n*th root of unity,

$$\begin{aligned} \sum _{k=0}^{n-1} (q;q^2)_k(-1)^kq^{-k^2} = \frac{(-q)_{n-1}}{(q)_{n}} \sum _{k=0}^{n-1}(-1)^kq^{\frac{k(3k+1)}{2}} (1-q^{2k+1}) \sum _{j=-k}^k (-1)^j q^{-j^2}. \end{aligned}$$

When $q= \zeta _n,$ a primitive odd *n*th root of unity, we obtain

$$\begin{aligned} \sum _{k=0}^{n-1} (q;q^2)_k(-1)^kq^{-k^2}&= \lim _{q \rightarrow \zeta _n} \frac{1}{n(1-q^n)} \sum _{k=0}^{n-1}(-1)^kq^{\frac{k(3k+1)}{2}} (1-q^{2k+1}) \sum _{j=-k}^k (-1)^j q^{-j^2}\\  &=-\frac{q}{n^2} \frac{d}{dq}\Big |_{q = \zeta _n} \sum _{k=0}^{n-1}(-1)^kq^{\frac{k(3k+1)}{2}} (1-q^{2k+1}) \sum _{j=-k}^k (-1)^j q^{-j^2}\\  &=-\frac{1}{n^2} \sum _{k=-n}^{n-1} \sum _{j=-k}^k \text {sgn}(k) (k(3k+1)/2 - j^2)(-1)^{k+j}q^{k(3k+1)/2 - j^2}. \end{aligned}$$

Replacing *q* by $q^{-1}$ and using (1.7) gives the desired result. $\square$

## Proofs of Theorems 1.1–1.4

In this section we use the Bailey chain to prove the generalizations of the identities of Cohen and Bryson–Ono–Pitman–Rhoades contained in Theorems 1.1–1.4.

### Proof of Theorem 1.1

We begin by applying Lemma 2.2$m-1$ times to the Bailey pair in (2.8) for $m \ge 1.$ The result is the Bailey pair relative to *q*, 

4.1 $$\begin{aligned} \alpha _n = \frac{q^{(m+1)n^2+mn}(1-q^{2n+1})}{1-q} \sum _{j=-n}^n (-1)^jq^{-j(3j+1)/2} \end{aligned}$$

and

4.2 $$\begin{aligned} \beta _n =\beta _{n_{m}}&= \sum _{n_{m} \ge \cdots \ge n_1 \ge 0} \frac{q^{n_{m-1}^2 + n_{m-1} + \cdots + n_1^2+n_1}}{(q)_{n_{m} - n_{m-1}}\cdots (q)_{n_2-n_1}} \nonumber \\&= \frac{1}{(q)_{n_{m}}}\sum _{n_m \ge \cdots \ge n_1 \ge 0} (q)_{n_1} \prod _{i=1}^{m-1} q^{n_i^2+n_i} \begin{bmatrix} n_{i+1} \\ n_i \end{bmatrix}. \end{aligned}$$

Inserting this into (3.3) and arguing as in the proof of (1.4), we find that for a primitive *n*th root of unity *q*, 

4.3 $$\begin{aligned}&\sum _{n-1 \ge n_m \ge \cdots \ge n_1 \ge 0} (q)_{n_m}(q)_{n_1}q^{n_m+1}\prod _{i=1}^{m-1} q^{n_i^2+n_i} \begin{bmatrix} n_{i+1} \\ n_i \end{bmatrix} \nonumber \\  &\quad = -\frac{1}{n} \sum _{k=-n}^{n-1}\sum _{j=-k}^k \left( mk^2 + (m-1)k -j(3j+1)/2\right) q^{mk^2 + (m-1)k -j(3j+1)/2}(-1)^j. \end{aligned}$$

Next we apply Lemma 2.3$m-1$ times to the Bailey pair in (2.7). The result is the Bailey pair relative to *q*, 

4.4 $$\begin{aligned} \alpha _n = \frac{q^{-(m-1)n^2-mn}(1-q^{2n+1})}{1-q} \sum _{j=-n}^n (-1)^jq^{j(3j+1)/2} \end{aligned}$$

and

4.5 $$\begin{aligned} \beta _n =\beta _{n_{m}}&= (-1)^{n_m}q^{-\left( {\begin{array}{c}n_m+1\\ 2\end{array}}\right) - n_m}\sum _{n_{m} \ge \cdots \ge n_1 \ge 0} \frac{q^{-n_mn_{m-1} - n_{m-1} - \cdots - n_2n_1 - n_1}(-1)^{n_1}q^{\left( {\begin{array}{c}n_1+1\\ 2\end{array}}\right) }}{(q)_{n_{m} - n_{m-1}}\cdots (q)_{n_2-n_1}(q)_{n_1}} \nonumber \\&= \frac{(-1)^{n_m}q^{-\left( {\begin{array}{c}n_m+1\\ 2\end{array}}\right) - n_m}}{(q)_{n_{m}}}\sum _{n_m \ge \cdots \ge n_1 \ge 0} (-1)^{n_1}q^{\left( {\begin{array}{c}n_1+1\\ 2\end{array}}\right) } \prod _{i=1}^{m-1} q^{-n_in_{i+1} - n_i} \begin{bmatrix} n_{i+1} \\ n_i \end{bmatrix}. \end{aligned}$$

Inserting this in (3.3) and arguing as usual gives that for *q* a primitive *n*th root of unity,

$$\begin{aligned} \begin{aligned}&\sum _{n-1 \ge n_m \ge \cdots \ge n_1 \ge 0} (-1)^{n_m+n_1}q^{-\left( {\begin{array}{c}n_m+1\\ 2\end{array}}\right) +\left( {\begin{array}{c}n_1+1\\ 2\end{array}}\right) } (q)_{n_m}\prod _{i=1}^{m-1} q^{-n_in_{i+1} - n_i} \begin{bmatrix} n_{i+1} \\ n_i \end{bmatrix} \\  &\quad = -\frac{1}{n} \sum _{k=-n}^{n-1}\sum _{j=-k}^k \left( mk^2 + (m-1)k -j(3j+1)/2\right) q^{-mk^2 - (m-1)k + j(3j+1)/2}(-1)^j. \end{aligned} \end{aligned}$$

Now, in the above we let $q=1/q$ and invoke (1.7) along with the fact that

$$\begin{aligned} \begin{bmatrix} n \\ k \end{bmatrix}_{q^{-1}} = \begin{bmatrix} n \\ k \end{bmatrix} q^{k^2-nk}. \end{aligned}$$

We obtain that if *q* is a primitive *n*th root of unity, then

$$\begin{aligned} \begin{aligned}&\sum _{n-1 \ge n_m \ge \cdots \ge n_1 \ge 0} (-1)^{n_1}q^{-\left( {\begin{array}{c}n_1+1\\ 2\end{array}}\right) } (q)_{n_m}\prod _{i=1}^{m-1} q^{n_i^2+n_i} \begin{bmatrix} n_{i+1} \\ n_i \end{bmatrix} \\  &\quad = -\frac{1}{n} \sum _{k=-n}^{n-1}\sum _{j=-k}^k \left( mk^2 + (m-1)k -j(3j+1)/2\right) q^{(m+1)k^2 + mk - j(3j+1)/2}(-1)^j. \end{aligned} \end{aligned}$$

Comparing with (4.3) gives the result. $\square$

We now turn to the proof of Theorem 1.2, which follows a similar principle.

### Proof of Theorem 1.2

We start with the same Bailey pair as in the proof of Theorem 1.1, that is the $(\alpha _n,\beta _n)$ given in (4.1) and (4.2), but now we insert it into (3.6). The result is

$$\begin{aligned}&\sum _{n_m =0}^{n-1} (-q)_{n_m}(q^{1-n})_{n_m} q^{n_m+1} \frac{1}{(q)_{n_{m}}}\sum _{n_m \ge \cdots \ge n_1 \ge 0} (q)_{n_1} \prod _{i=1}^{m-1} q^{n_i^2+n_i} \begin{bmatrix} n_{i+1} \\ n_i \end{bmatrix} \\&\quad = -(-q)^{n}\frac{(-q)_{n-1}}{(q)_{n}}\sum _{k=0}^{n-1} \frac{(q^{1-n})_k}{(q^{1+n})_k}(-1)^k q^{nk+mk^2+(m-1)k}(1-q^{2k+1}) \sum _{j=-k}^k (-1)^jq^{-j(3j+1)/2}. \end{aligned}$$

Arguing as usual, we get that for a primitive even *n*th root of unity *q*, 

4.6 $$\begin{aligned}&\sum _{n-1 \ge n_m \ge \cdots \ge n_1 \ge 0} (-q)_{n_m} (q)_{n_1} q^{n_m+1} \prod _{i=1}^{m-1} q^{n_i^2+n_i} \begin{bmatrix} n_{i+1} \\ n_i \end{bmatrix} \nonumber \\&\quad = \lim _{q \rightarrow \zeta _n} \frac{-(-q)_{n-1}}{n(1-q^n)} \sum _{k=0}^{n-1}\sum _{j=-k}^k (-1)^{j+k}q^{mk^2+(m-1)k-j(3j+1)/2}(1-q^{2k+1}) \nonumber \\&\quad = \frac{1}{4} \sum _{k=-n}^{n-1}\sum _{j=-k}^k \textrm{sgn}(k)(-1)^{j+k}q^{mk^2+(m-1)k-j(3j+1)/2}. \end{aligned}$$

Now we take the Bailey pair of (4.4) and (4.5) and insert it into (3.6). This gives

$$\begin{aligned}&\sum _{n-1 \ge n_m \ge \cdots \ge n_1 \ge 0} (-q)_{n_m} (-1)^{n_1+n_m} (q^{1-n})_{n_m} \frac{q^{1-\left( {\begin{array}{c}n_m+1\\ 2\end{array}}\right) +\left( {\begin{array}{c}n_1+1\\ 2\end{array}}\right) }}{(q)_{n_{m}}} \prod _{i=1}^{m-1} q^{-n_in_{i+1} - n_i} \begin{bmatrix} n_{i+1} \\ n_i \end{bmatrix} \\&\quad = -(-q)^{n}\frac{(-q)_{n-1}}{(q)_{n}}\sum _{k=0}^{n-1} \frac{(q^{1-n})_k}{(q^{1+n})_k}(-1)^kq^{nk - m k^2 -(m+1)k}(1-q^{2k+1}) \sum _{j=-k}^k (-1)^jq^{j(3j+1)/2}. \end{aligned}$$

Dividing both sides by *q* and rearranging, we obtain that for a primitive even *n*th root of unity *q*, 

$$\begin{aligned}&\sum _{n-1 \ge n_m \ge \cdots \ge n_1 \ge 0} (-q)_{n_m} (-1)^{n_1+n_m} q^{-\left( {\begin{array}{c}n_m+1\\ 2\end{array}}\right) +\left( {\begin{array}{c}n_1+1\\ 2\end{array}}\right) } \prod _{i=1}^{m-1} q^{-n_in_{i+1} - n_i} \begin{bmatrix} n_{i+1} \\ n_i \end{bmatrix} \\&\quad = \lim _{q \rightarrow \zeta _n} \frac{(-q)_{n-1}}{n(1-q^n)} \sum _{k=0}^{n-1} \sum _{j=-k}^k (-1)^kq^{- m k^2 -(m-1)k}(1-q^{-(2k+1)}) (-1)^jq^{j(3j+1)/2} \nonumber \\&\quad = -\frac{1}{4} \sum _{k=-n}^{n-1}\sum _{j=-k}^k \textrm{sgn}(k)(-1)^{j+k}q^{-mk^2-(m-1)k+j(3j+1)/2}. \end{aligned}$$

Letting $q=1/q$ in the above gives

$$\begin{aligned} \begin{aligned}&\sum _{n-1 \ge n_m \ge \cdots \ge n_1 \ge 0} (-q)_{n_m} (-1)^{n_1+n_m} q^{-\left( {\begin{array}{c}n_1+1\\ 2\end{array}}\right) } \prod _{i=1}^{m-1} q^{n_i^2+n_i} \begin{bmatrix} n_{i+1} \\ n_i \end{bmatrix} \\&\quad = -\frac{1}{4} \sum _{k=-n}^{n-1}\sum _{j=-k}^k \textrm{sgn}(k)(-1)^{j+k}q^{mk^2+(m-1)k-j(3j+1)/2}. \end{aligned} \end{aligned}$$

Comparing with (4.6) now gives the result. $\square$

### Proof of Theorem 1.3

We begin by applying Lemma 2.2$m-1$ times to the Bailey pair in (2.9) for $m \ge 1.$ The result is the Bailey pair relative to *q*, 

$$\begin{aligned} \alpha _n = \frac{q^{n(3n+1)/2 + (m-1)(n^2+n)}(1-q^{2n+1})}{1-q}\sum _{j=-n}^n(-1)^jq^{-j^2} \end{aligned}$$

and

$$\begin{aligned} \beta _n = \beta _{n_m} = \frac{1}{(q)_{n_m}}\sum _{n_m \ge \cdots \ge n_1 \ge 0} \frac{(q)_{n_1}}{(-q)_{n_1}}\prod _{i=1}^{m-1} q^{n_i^2+n_i} \begin{bmatrix} n_{i+1} \\ n_i \end{bmatrix}. \end{aligned}$$

Inserting this into (3.9), letting *q* be a primitive *n*th root of unity, and calculating as usual, we obtain

4.7 $$\begin{aligned}&\sum _{n-1 \ge n_m \ge \cdots \ge n_1 \ge 0} (-q^{n_1+1})_{n_m-n_1}(-1)^{n_m}(q)_{n_1}\prod _{i=1}^{m-1} q^{n_i^2+n_i} \begin{bmatrix} n_{i+1} \\ n_i \end{bmatrix} \nonumber \\  &\quad = {\left\{ \begin{array}{ll} -\frac{1}{n^2} \mathop {\mathop {\sum }\limits _{{k=-n}}}\limits ^{{n-1}} \mathop {\mathop {\sum }\limits _{{j=-k}}}\limits ^{k} \text {sgn}(k) (k(3k+1)/2 +(m-1)(k^2+k) - j^2)\\ \qquad \qquad \qquad \times (-1)^{k+j}q^{k(3k+1)/2 + (m-1)(k^2+k)- j^2}, \qquad n \text{ odd } , \\ \frac{1}{4} \mathop {\mathop {\sum }\limits _{{k=-n}}}\limits ^{{n-1}} \mathop {\mathop {\sum }\limits _{{j=-k}}}\limits ^{k} \text {sgn}(k)(-1)^{k+j}q^{k(3k+1)/2 +(m-1)(k^2+k)- j^2}, \ \ n \text{ even } . \end{array}\right. } \end{aligned}$$

Note that for $m > 1$ the multisum on the left-hand side above does not truncate at odd roots of unity without the upper bound $n-1.$

Next we let $q=q^2$ and apply Lemma 2.2$m-1$ times to the Bailey pair in (2.10). The result is the Bailey pair relative to $q^2,$

$$\begin{aligned} \alpha _n = \frac{q^{2mn^2+(2m-1)n}(1-q^{2n+1})}{1-q}\sum _{j=-n}^n (-1)^jq^{-j^2} \end{aligned}$$

and

$$\begin{aligned} \beta _n = \beta _{n_m} = \frac{1}{(q^2;q^2)_{n_m}}\sum _{n_m \ge \cdots \ge n_1 \ge 0} \frac{(q^2;q^2)_{n_1}}{(-q^2)_{2n_1}} \prod _{i=1}^{m-1} q^{2n_i^2+2n_i} \begin{bmatrix} n_{i+1} \\ n_i \end{bmatrix}_{q^2}. \end{aligned}$$

Inserting this pair into Lemma 2.7, we obtain

$$\begin{aligned} \begin{aligned}&\sum _{n-1 \ge n_m \ge \cdots \ge n_1 \ge 0} \frac{(-q^{2n_1+2})_{2n_m-2n_1}(q^{2-2n};q^2)_{n_m}(-1)^{n_m}(q^2;q^2)_{n_1}}{(q^2;q^2)_{n_m}} \\&\qquad \times q^{n(2n_m+1)-(n_m+1)^2} \prod _{i=1}^{m-1} q^{2n_i^2+2n_i} \begin{bmatrix} n_{i+1} \\ n_i \end{bmatrix}_{q^2} \\&\quad = \frac{(-q)_{n-1}}{(q)_{n}}\sum _{k=0}^{n-1} \sum _{j=-k}^k \frac{(q^{1-n})_k}{(q^{1+n})_k}(-1)^{k+j}q^{nk +k(3k+1)/2 +(2m-2)(k^2+k)-j^2}(1-q^{2k+1}). \end{aligned} \end{aligned}$$

Now we wish to let *q* be a primitive *n*th root of unity. On the left-hand side, if *n* is odd, then $q^2$ is also a primitive *n*th root of unity and so

$$\begin{aligned} \frac{(q^{2-2n};q^2)_{n_m}}{(q^2;q^2)_{n_m}} = 1. \end{aligned}$$

If *n* is even, then this term takes the form 0/0 when $n_m \ge n/2.$ But then either $n_1 \ge n/2,$ in which case $(q^2;q^2)_{n_1} = 0,$ or $n_1 < n/2,$ in which case $(-q^{2n_1+2})_{2n_m - 2n_1} = 0.$ So this is never an issue (and in fact, the sum actually truncates at $n/2-1$). The right-hand side evaluates as usual, and we obtain

4.8 $$\begin{aligned}&\sum _{n-1 \ge n_m \ge \cdots \ge n_1 \ge 0} (-q^{2n_1+2})_{2n_m-2n_1}(-1)^{n_m}q^{-(n_m+1)^2}{(q^2;q^2)_{n_1}} \prod _{i=1}^{m-1} q^{2n_i^2+2n_i} \begin{bmatrix} n_{i+1} \\ n_i \end{bmatrix}_{q^2} \nonumber \\  &\quad = {\left\{ \begin{array}{ll} -\frac{1}{n^2} \mathop {\mathop {\sum }\limits _{{k=-n}}}\limits ^{{n-1}} \mathop {\mathop {\sum }\limits _{{j=-k}}}\limits ^{k} \text {sgn}(k) (k(3k+1)/2 +(2m-2)(k^2+k) - j^2) \\ \qquad \qquad \qquad \times (-1)^{k+j}q^{k(3k+1)/2 + (2m-2)(k^2+k)- j^2}, \qquad n \text{ odd } , \\ \frac{1}{4} \mathop {\mathop {\sum }\limits _{{k=-n}}}\limits ^{{n-1}} \mathop {\mathop {\sum }\limits _{{j=-k}}}\limits ^{k} \text {sgn}(k)(-1)^{k+j}q^{k(3k+1)/2 +(2m-2)(k^2+k)- j^2}, \ \ n \text{ even } . \end{array}\right. } \end{aligned}$$

Comparing this with (4.7) at $m \mapsto 2m-1$ gives the result. $\square$

### Proof of Theorem 1.4

Apply Lemma 2.2$m-1$ times with $q=q^2$ to the Bailey pair in (2.11). We obtain the following Bailey pair relative to $q^2$:

$$\begin{aligned} \alpha _n = \frac{q^{2n^2}(1-q^{4n+2})}{1-q^2}\sum _{j=-n}^n (-1)^jq^{-j^2} \end{aligned}$$

and

$$\begin{aligned} \beta _n = \beta _{n_m} = \frac{1}{(q^2;q^2)_{n_m}}\sum _{n_m \ge \cdots \ge n_1 \ge 0} \frac{(q;q^2)_{n_1}}{(-q)_{2n_1}} \prod _{i=1}^{m-1} q^{2n_i^2+2n_i} \begin{bmatrix} n_{i+1} \\ n_i \end{bmatrix}_{q^2}. \end{aligned}$$

Inserting this pair into Lemma 2.8, and taking *q* to be a primitive *n*th odd root of unity leads to

$$\begin{aligned} \begin{aligned}&\sum _{n-1 \ge n_m \ge \cdots \ge n_1 \ge 0} (-q^{2n_1+1})_{2n_m-2n_1}(-1)^{n_m}q^{-n_m^2}(q;q^2)_{n_1} \prod _{i=1}^{m-1} q^{2n_i^2+2n_i} \begin{bmatrix} n_{i+1} \\ n_i \end{bmatrix}_{q^2}\\&\quad =\frac{(-q)_{n-1}}{(q)_{n}}\sum _{k=0}^{n-1} \sum _{j=-k}^k (-1)^{k+j}q^{k(3k+1)/2 +(2m-2)(k^2+k)-j^2}(1-q^{2k+1}). \end{aligned} \end{aligned}$$

Arguing as usual gives

$$\begin{aligned} \begin{aligned}&\sum _{n-1 \ge n_m \ge \cdots \ge n_1 \ge 0} (-q^{2n_1+1})_{2n_m-2n_1}(-1)^{n_m}q^{-n_m^2}(q;q^2)_{n_1} \prod _{i=1}^{m-1} q^{2n_i^2+2n_i} \begin{bmatrix} n_{i+1} \\ n_i \end{bmatrix}_{q^2}\\  &\quad =-\frac{1}{n^2} \sum _{k=-n}^{n-1} \sum _{j=-k}^k \text {sgn}(k) (k(3k+1)/2 +(2m-2)(k^2+k) - j^2)\\  &\qquad \times (-1)^{k+j}q^{k(3k+1)/2 + (2m-2)(k^2+k)- j^2}. \end{aligned} \end{aligned}$$

Comparing this with (4.8) gives the result. $\square$

## Concluding remarks

In this paper we have shown how classical *q*-series identities at roots of unity can be proved using the Bailey pair machinery. Applying Bailey pairs developed in the study of mock theta functions to the classical Bailey lemma and some base changing Bailey lemmas allows us to express the relevant *q*-series at roots of unity as truncated indefinite binary theta functions, and then comparing these expressions gives the results. Understanding the identities in the context of Bailey pairs leads to an effortless generalization to infinite families via the Bailey chain.

In future papers we intend to develop this work in at least two directions. First, expanding both the arsenal of Bailey pairs and the specializations of the Bailey lemma will give many new quantum *q*-series identities. Second, in many instances the expressions for the *q*-series at roots of unity will involve truncated unary theta functions, and in joint work with Amanda Folsom we plan to explore the implications of such expressions for the quantum modularity of the corresponding *q*-series.

## Data Availability

Not applicable.

## References

[1] Andrews, G.E.: Multiple series Rogers–Ramanujan type identities. Pac. J. Math. **114**(2), 267–283 (1984)

[2] Andrews, G.E.: The fifth and seventh order mock theta functions. Trans. Am. Math. Soc. **293**(1), 113–134 (1986)

[3] Andrews, G.E.: Bailey’s transform, lemma, chains and tree. In: Special Functions 2000: Current Perspective and Future Directions (Tempe, AZ). NATO Science Series II: Mathematics, Physics and Chemistry, edited by Joaquin Bustoz, Mourad E. H. Ismail and Sergei K. Suslov, vol. 30, pp. 1–22. Kluwer Acad. Publ., Dordrecht (2001)

[4] Andrews, G.E.; Hickerson, D.: Ramanujan’s “lost’’ notebook. VII. The sixth order mock theta functions. Adv. Math. **89**(1), 60–105 (1991)

[5] Bressoud, D.; Ismail, M.E.H.; Stanton, D.: Change of base in Bailey pairs. Ramanujan J. **4**(4), 435–453 (2000)

[6] Bryson, J.; Ono, K.; Pitman, S.; Rhoades, R.C.: Unimodal sequences and quantum and mock modular forms. Proc. Natl. Acad. Sci. USA **109**(40), 16063–16067 (2012)

[7] Cohen, H.: -identities for Maass waveforms. Invent. Math. **91**(3), 409–422 (1988)

[8] Hikami, K.; Lovejoy, J.: Torus knots and quantum modular forms. Res. Math. Sci. **2**, 2 (2015)

[9] Lovejoy, J.: Lacunary partition functions. Math. Res. Lett. **9**, 191–198 (2002)

[10] Lovejoy, J.: Bailey pairs and strange identities. J. Korean Math. Soc. **59**(5), 1015–1045 (2022)

[11] Lovejoy, J.: Quantum -series identities. Hardy-Ramanujan J. (Special Commemorative Volume in Honour of Srinivasa Ramanujan) **44**, 61–73 (2021)

[12] Lovejoy, J.; Osburn, R.: The colored Jones polynomial and Kontsevich–Zagier series for double twist knots, II. N. Y. J. Math. **25**, 1312–1349 (2019)

[13] Lovejoy, J.; Osburn, R.: The colored Jones polynomial and Kontsevich–Zagier series for double twist knots. J. Knot Theory Ramif. **30**, 2150031 (2021)

[14] Warnaar, S.O.: 50 years of Bailey’s lemma. In: Algebraic Combinatorics and Applications (Gößweinstein, 1999), edited by Anton Betten, Axel Kohnert, Reinhard Laue and Alfred Wassermann, pp. 333–347. Springer, Berlin (2001)

